# Achieving optimal heath data impact in rural African healthcare settings: measures to barriers in Bukomansimbi District, Central Uganda

**DOI:** 10.1186/s12939-022-01814-1

**Published:** 2022-12-28

**Authors:** Chraish Miiro, Josephine Caren Ndawula, Enoch Musudo, Olivia Peace Nabuuma, Charles Norman Mpaata, Shamim Nabukenya, Alex Akaka, Olivia Bebembeire, Douglas Sanya

**Affiliations:** 1 Department of Pharmacy, Makerere University, Kampala, 7072, Uganda; 2 School of Medicine, Makerere University, Kampala, 7072, Uganda; 3 School of Health Sciences, Makerere University, Kampala, 7072, Uganda

**Keywords:** Health data, Healthcare professionals, Qualitative research

## Abstract

**Background:**

Health data is one of the most valuable assets in health service delivery yet one of the most underutilized in especially low-income countries. Health data is postulated to improve health service delivery through availing avenues for optimal patient management, facility management, and public health surveillance and management. Advancements in information technology (IT) will further increase the value of data, but will also call for capacity readiness especially in rural health facilities.

We aimed to understand the current knowledge, attitudes and practices of health workers towards health data management and utilization.

**Methods:**

We conducted key informant interviews (KII) for health workers and data staff, and focus group discussions (FGD) for the village health teams (VHTs). We used both purposive and convenience sampling to recruit key informants, and convenience sampling to recruit village health teams. Interviews and discussions were audiotaped and transcribed verbatim. We manually generated the codes and we used thematic analysis to identify the themes. We also developed a reflexivity journal.

**Results:**

We conducted a total of 6 key informant interviews and 3 focus group discussions of 29 participants. Our analysis identified 7 themes: One theme underscored the health workers’ enthusiasm towards an optimal health data management setting. The rest of the six themes resonated around working remedies to the systemic challenges that grapple health data management and utilization at facilities in rural areas. These include: Building human resource capacity; Equipping the facilities; Improved coordination with partners; Improved data quality assurance; Promotion of a pull supply system and Reducing information relay time.

**Conclusion:**

Our findings reveal a plethora of systematic challenges that have persistently undercut optimal routine health data management and utilization in rural areas and suggest possible working remedies. Health care workers express enthusiasm towards an optimal health management system but this isn’t matched by their technical capacity, facility readiness, systems and policy willingness. There is an urgent need to build rural lower facilities’ capacity in health data management and utilization which will also lay a foundation for exploitation of information technology in health.

## Background

Health data is one of the most valuable assets in health service delivery. Data informs patient clinical interventions, facility management and public health managementby offering the basis upon which decisions are made [2]. However, this asset hasn’t been put to good use in most developing countries like Uganda [38, 5]. As the healthcare outset still grapples with human resource [44], equipment and infrastructure shortages, health data has been the unfortunate opportunity cost especially in rural settings [27, 46].

The ministry of health of Uganda together with stakeholders and development partners adapted the Health Management Information system (HMIS) as an integrated reporting system to collect relevant and functional information on a routine basis in 1993 [11]). Data governance was decentralized through creating the District Health Information Systems I and later II(DHIS I and DHIS II) to govern HMIS data in each respective district [26].

With advancements in information computer technology (ICT), plans were devised to transfer medical records into an Electronic Medical Records System(EMRS). The Open MRS system was introduced in 2009 and rolled out in HIV and TB care. All data in HIV and TB care were transferred into the Open MRS and there is a plan to roll out an electronic system in the entirety of health service delivery points. Subsequently, Uganda launched her National eHealth Policy and Strategy in May 2018 to guide and deliver the digital health information age [24].

The HMIS system has been in force despite being a highly laborious and tedious physical process, lacking easy archival and retrieval means and posing significant logistical challenges [13, 28]. Consequent discrepancies in the DHIS II like missing variables, inaccuracy, inconsistency and incompleteness have been identified in various districts. All these have continuously retarded the valuable potential of the health information [16, 13].

Electronic MRS may even face a worse doom than paper based HMIS given that it requires more structural and human resource input than the latter [6]. With shortages of both infrastructure and human resources.

The end goal is to have an easily usable, archivable and retrievable data management system that will inform patient clinical management, facility management, public health, district and national resource allocation in that order [17, 32, 43]. However, paper based HMIS system has consistently come short of these expectations and so will the EMRS if current challenges are not identified and solved.

Rural Lower-level health facilities form the single biggest constituent of public health service in Uganda and most other Low-Income Countries (LICs), therefore, any effort in attaining a good Health Records System will have to favorably consider them. Establishing the current status of data management through the lens of data handlers will be imperative in identifying gaps to fill, and facilitators to promote [1, 12, 32].

Any health data management program will face the current fate, if prevailing challenges are not identified and solutions devised. We set out to qualitatively analyze the current status of health data management at Mirambi Health Centre III, with keen interest in data collection, compilation, analysis, synthesis and dissemination.

## Methods

### Research design

This was a qualitative, cross-sectional, field-based study in which health data access and utilisation behaviours and practices of healthcare personnel and village health teams at facility and district level were studied/assessed.

### Research site and settings

Mirambi Health Centre(HC) III is in a rural setting, located 147kms South West of Kampala, the Ugandan Capital, and 20kms north of nearby Masaka City. It has both the inpatient and outpatient departments and a functional laboratory. It offers antenatal care, treats common diseases, conducts immunization and outreach services on top of environmental health. It has 13 staff led by a clinical officer. The facility is projected to serve around 20,000 members of the community just as any HCIII [25]. The Bukomansimbi District Health Office (DHO) is located at the district headquarters, 10 miles away from Mirambi HC III. It shared an incomplete structural hall with other district administrative units [8].

### Sampling procedure and recruitment

Purposive and convenience sampling were employed to identify the key informants. We invited the key informants to participate in the study through phone calls, emails and visits at the facilities. In total, we recruited 6 key informants, 4 from Mirambi HC III, and 2 from the DHO. For the focus group discussions participants, all VHTs centered at Mirambi HC III we recruited. They were drawn from the parishes of Mirambi and Kiryasaaka. They were invited through the HC in liaison with the VHT leaders, and made necessary reminders using megaphones, community radio and phone calls.

### Data collection

Qualitative methods were mainly employed, coupled with guiding observations around the facility. VHTs from each parish were grouped into two groups, giving us a total of 4 focus group discussions of 8, 8, 8 and 5 participants respectively [21]. We conducted a total of 6 key informant interviews, 4 from the HC III, and 2 from the DHO. We interacted with clinicians, a senior administrator and 2 health data focal personnel.

The open-ended questions focused on exploring the following:

- The competence in the steps of the health data flow chain.
- The attitudes towards health data management.
- The current practices in health data management, specifically probing the utilization.
- The facilitators and limitations of health data flow chain from the patient/community to the national datasets.

We also made necessary observations and took pictures for guidance during analysis.

### Data analysis

We deployed both semantic and latent thematic analysis approaches to the data. We adopted Braun and Clarke’s framework [42]. The audios were transcribed verbatim, and codes were manually generated from the transcripts. The coding process was deductively guided by the two interview guides, which referenced the primary research questions and objectives. However, spontaneous codes that were realized outside the primary guide were considered. Five research team members (NOP, NS, ME, AA, and MC) participated in the coding process and MC went over each script at least twice to exhaust relevant codes.

Themes were developed through aggregating similar or related codes, promotion of some prominent codes, and alteration of predicted and realized themes. The reflexivity journal attained shape after the 2^nd^ round of coding, and was edited as more codes and themes were identified.

There were 6 rounds of coding and theme development as more meditation was needed to capture underlying ideas.

## Results

### Participant characteristics

We conducted a total of 6 key informant interviews and 4 focus group discussions (FGDs) of 8(FGD1), 8(FGD2), 8(FGD3) and 5(FGD4) participants each (Table 1). 2 key informants were data people, 1 was an assistant district health officer, 1 was a laboratory technologist and 2 were nursing officers. FGDs involved only VHTs without their leaders.

**Table 1:** Participant Characteristics

	**Healthcare Workers**	**VHTs**	**Data Staff**
**Gender**			
Male	**3(75%)**	**9(31%)**	**1(50%)**
Female	**1(25%)**	**20(69%)**	**1(50%)**
**Interview type**			
KII	**4(100%)**	**-**	**2(100%)**
FGD	**-**	**29(100%)**	**-**

### We identified 7 themes from the 27 codes we generated (Table 2)

**Table 2:** Themes, Codes And Supporting Verbatim

THEMES	CODES	VERBATIM
Facility Staff and VHTs are positively receptive of an optimal health data management and utilization process and underscore the value therein	Healthcare workers and VHTs understand what health data is, and they underscore its value in optimal healthcare delivery	“Health data is the data or information that we collect from health-related activities. This can include the age, sex, weight of the person and they disease they are suffering from.” FGD1 “Captures data about disease incidence rate.” KII3 “Captures patient medical and drug history for future reference” KII3 “Data can help anticipate need” FGD3 “This data helps in emergency mitigation” FGD2 “This data helps us when we are referring patients” FGD3
	The facility staff indicated knowing how to use the respective HMIS tools available at their disposal in the respective departments, and they routinely collected the data in physical form	“I use specific HMIS book to capture lab records. Each department has a specific book.” KII1 “There are different HMIS books they use like 105, 33B etc.” KII4
	Staff and VHTs improvise to ensure health data is collected	“Sometimes we buy books where we enter the data when we run out of HMIS tools. We transfer this data when the registers arrive.” KII1
There is a need to develop and implement a robust, elaborate and standard training module in the core aspects of data management (collection, handling and utilization) for the data focal personnel and other healthcare staff	The data training was deemed inadequate, and a more standardized approach was advocated for	Facility staff are computer literates but not to a level of using basic analytic tools. This includes focal data personnel at the facility “Make the certificate of 2 years the minimal standard of recruitment so they improve the skills of the data personnel.” KII4 “Avail farther training and mentorships.” KII1 “There might be a knowledge gap in data utilization.” KII2 “People don’t know the idea of comparing trends.” KII1 “The data personnel are trained, but their trainers are also insufficient. I think they learn more on job and the good thing they ask “what is this?” (Referring to indictors) and we teach them here” KII1 **“The training we receive is not enough”**
	VHTs requested for more contact time, for training. Also, interactions come with financial benefits for them	“The training we receive is not enough” FGD1 “They should increase the frequency of training” FGD2 “We have training meetings every quarter at the district where they give us a small allowance if we attend” FGD3
Develop or adopt realistic data quality control protocols to fit all facility data processes	There is minimal coordination between healthcare personnel and data staff	“The HMIS focal person picks the data from each department and submits it to the HCIV or the district without any discussion with the departments about the data picked” KII4
	There is minimal supervision of data in each department	There is supposed to be data cleaning, then verification etc., but these may all be skipped “I don’t supervise data management in my department” KII1 “I have never compared trends and am not so sure it is done at the district” KII3
	Performance evaluation is more quantitative than qualitative	“The implementing partner sets a target of like 10 positives per day.” KII 1
Create a conducive workspace in terms of facilities, equipment, staffing and renumeration	The facility lacks a data office to support the data flow processes	No organized data room Rudimentary means of data recording “We don’t have/use a computer currently” (KII1) “From Mirambi, you have to go to Masaka to make a photocopy since the facility lacks a photocopier”(KII4)
	There is insufficient human resource capacity to optimally execute data management at the facility	Understaffing “We are critically understaffed at this facility”KII1 “Work is overwhelming: I am supposed to work for only 3 days a week yet I end up working for 5 days and no one pays for the extra 2 days” KII2 “At some point, both midwives got maternity leave at the same time” KII1
	VHTs argued that appropriate incentives will improve their overall output, especially in data collection and utilization	“We need to be prioritized for healthcare services to motivate us.” FGD1 “They should give us free health services” FGD 4 “VHTs are not prioritized while receiving treatment at the facility” FGD2 “We need a standard allowance for data collection that should be paid in time.” FGD 3 “Our allowance is very small, and yet again it delays.” FGD 4
The pull supply system would both optimize routine data utilization and reduce associated shortages	Existing procurement and resource allocation policies don’t exploit health data thus hindering capacity building among staff	“We make orders basing on the data we have but NMS and JMS supply what they have.” KII 3 “Allocation of resources is at a higher level. Therefore, knowing all this (having datasets) may not help the decisions that are made at a higher level.” KII 1
	Limited supplies are exposing VHTs to poor working conditions that totally compromise their output in the data chain	VHTs lack wet season gear “It’s hard to do home visits in the rain season because we don’t have rain coats, gumboots and umbrellas” FGD 2 “We need torches, umbrellas and gumboots” FGD 3 “The bicycles they gave us no longer work” FGD 1 “The villages are so big; we may end up walking long distances” FGD1
	Shortages in tools and other supplies increase the data workload	“We occasionally run out of registers and we always improvise. When the registers come, those who can, transfer this information but this essentially doubles our workload.” KII1 “Sometimes they bring us the tools abruptly, they call us and then force us to work under extreme pressure” FGD3
	The facility liaises with other public health centres to overcome some shortages	“We usually borrow registers from other HCs if they have.” KII5
There is a need for improved coordination between the facility and its implementation partners to create harmony in health data management	The facility and respective implementing partners run parallel recruitment and training schedules	“Implementing partners recruit data staff on short contract basis. So, the staff are not motivated after all the contracts are to expire soon.” KII5 “We received training from Mildmay at Brovard Hotel, and from Rakai at the District.” KII2 “Each time they bring a new trainer” KII6
	Quality control, and M&E protocols implemented by the facility are divergent from those implemented by partners	“The implementing partner sets a target of like 10 positives per day.” KII 1 “Funders demands(targets) give us so much pressure.” KII5 “You may be pushed to force a positive to hit the targets or else, the implementing partner will ask questions” KII1
	Health data from private health units is mostly missed	Under reporting especially from local drug shops, TBAs, and illegal HC structures” KII4 “There are many private HCs that don’t report.”KII6 “Some health structures operate illegally so we can’t collect their data” KII4
Reduction in information relay time, and improved community engagement will optimize the impact of health data utilization	Data dissemination is inadequately funded	“There is a plan to warn communities about seasonal diseases but it is not funded. The program is therefore implemented at just around 50%”. (KII4)
	There is a big time-lag between data collection and expected utilization. The local relevance of the data is mostly lost to time	“There are quarterly assessments and comparisons.” KII6 “There’s no local analysis and therefore results are only relayed back after the district quarterly meetings.” KII1 Therefore, feedback from data collected can come as late as 6 months
	Information is not relayed back to the communities where data is collected. The communities get bored of the monotonous data collection routine without feedback	“Data is not given back to people by health organizations that collect it” FGD 1 “We revisit the communities every after 3 months but people get tired of us” FGD2 “We are not given feedback about the research projects we partake in.” FGD3 “We are not given explanations for failed programmes, so we also can’t explain to community members.” FGD3
	VHTs need support from healthcare workers during community engagement	“Relaying information to the public needs a new face, not only the usual VHTs” FGD3 “People are so adopted to the VHTs that it is becoming hard to collect data” FGD3 “Some people think we don’t have capacity to do our work” FGD2
	Community members have economic expectations from home visits	“Some community members think we are paid a lot of money and we don’t share with them.” FGD2 “People think we are supposed to give them something each time we visit them, eventually they chase us since we don’t give anything.” FGD4
	Political affiliations influence health information relay to the community	“Some people think we have political intentions so they don’t welcome us” FGD2 “Some people believe we are campaigning for the government so they resent us” FGD3

1Makerere University Pharmacy Department, P.O. Box 7072 Kampala, Uganda

2Makerere University School of Medicine, P.O. Box 7072 Kampala, Uganda

3Makerere University School of Biomedical Sciences, P.O. Box 7072 Kampala, Uganda

4Makerere University School of Public Health, P.O. Box 7072 Kampala, Uganda

1. Facility Staff and VHTs are positively receptive of an optimal health data management and utilization process; 2. There is a need to develop a robust, elaborate and standard training module in the core aspects of data management (collection, handling and utilization) for the data focal personnel and other healthcare staff. 3. Develop or adopt realistic data quality control protocols to fit all facility data processes. 4. Create a conducive workspace in terms of facilities, equipment, staffing and renumeration. 5. The pull supply system would both optimize routine data utilization and reduce associated shortages. 6. There is a need for improved coordination between the facility and its implementation partners to create harmony in health data management; 7. Reduction in information relay time, and improved community engagement will optimize the impact of health data utilization.

The following codes supported the above themes.

### Healthcare workers’ and VHTs’ understanding of health data

Healthcare workers and VHTs understood what health data is, and they underscored its value in optimal healthcare delivery.

“Health data is the data or information that we collect from health-related activities. This can include the age, sex, weight of the person and they disease they are suffering from”.FGD1.

“Captures data about disease incidence rate.” KII3.

“Captures patient medical and drug history for future reference” KII3.

“Data can help anticipate need” FGD1.

“This data helps in disease prevention” FGD1.

“Helps inform medicine needs for the village” FGD2.

“This data helps in immunization planning” FGD3.

“This data helps us when we are referring patients” FGD3.

### Improvision in data collection

Facility staff and VHTs undertook improvision initiatives to ensure routine health data collection amidst the shortages. This signifies the value they attach to routine health data collection.

“Sometimes we buy books where we enter the data when we run out of HMIS tools. We transfer this data when the registers arrive”KII1.

“We buy exercise books when the registers delay”FGD2.

### Training in data management for facility staff

The data training was deemed inadequate, and a more standardized approach was advocated for. Most staff admitted to never receiving any training regarding data analysis and translation into workable forms. “Make the certificate of 2 years the minimal standard of recruitment so they improve the skills of the data personnel.” KII4.

“Avail farther training and mentorships.” KII1.

“There might be a knowledge gap in data utilization.” KII2.

“People don’t know the idea of comparing trends.”KII1.

“The data personnel are trained, but their trainers are also insufficient. I think they learn more on job and the good thing they ask “what is this?” (Referring to indictors) and we teach them here” KII1.

### VHTs’ training in data management

VHTs requested for more training contact time to improve their skillsets and maximize training associated amenities like allowances and good food.

“The training we receive is not enough” FGD1.

“They should increase the frequency of training” FGD2.

“We have training meetings every quarter at the district where they give us a small allowance if we attend” FGD3.

### Interdepartmental cooperation

There is minimal deliberation on collected data between the clinical, laboratory, pharmacy and the data team.

“The HMIS focal person picks the data from each department and submits it to the district without any discussion with the departments about the data picked” KII4.

### Human resource capacity

The facility has human resource gaps. This stretches the available staff and so they commit less time and effort to “non-clinical” aspects of work like proper data collection, management and utilization [8].

“We are critically understaffed at this facility”KII1.

“Work is overwhelming: I am supposed to work for only 3 days a week yet I end up working for 5 days and no one pays for the extra 2 days” KII2.

“At some point, both midwives got maternity leave at the same time” KII1.

### Collaboration between clinical personnel and data staff

There is minimal coordination between healthcare personnel and data staff.

“The HMIS focal person picks the data from each department and submits it to the HCIV or the district without any discussion with the departments about the data picked” KII4.

### Departmental level data supervision

There is minimal supervision of data management in each department. Data cleaning and verification are overlooked and this creates a Garbage in Garbage out situation with HMIS data.

“I don’t supervise data management in my department” KII1.

“I have never compared trends and am not so sure it is done at the district” KII3.

### Performance evaluation

Performance evaluation overlooks qualitative aspects of data, yet unrealistic quantitative parameters like specific number of positive RDTs per day could take a toll on the authenticity of the data.

“The implementing partner sets a target of like 10 positives per day.” KII 1.

### Facilities and equipment

The facility lacks a data office, and equipment to support the data flow processes. Data was mostly kept in physical form at the facility, till submitted to the district for interpretation and then relayed back to the facility:

“We don’t have/use a computer currently” (KII1).

“From Mirambi, you have to go to Masaka to make a photocopy since the facility lacks a photocopier”(KII4).

### Working conditions

Limited supplies are exposing VHTs to poor working conditions that totally compromise their output in the data chain. Each and every VHT member complained of shortages in transport means, equipment for bad weather protection and lighting in the night.

“It’s hard to do home visits in the rain season because we don’t have rain coats, gumboots and umbrellas” FGD 2.

“We need torches, umbrellas and gumboots” FGD 3.

“The bicycles they gave us no longer work” FGD 1.

“The villages are so big; we may end up walking long distances” FGD1.

### Data collection incentives

VHTs argued that appropriate incentives will improve their overall output, especially in data collection and utilization.

“We need to be prioritized for healthcare services to motivate us.” FGD1.

“They should give us free health services” FGD 4.

“VHTs are not prioritized while receiving treatment at the facility” FGD2.

“We need a standard allowance for data collection that should be paid in time.” FGD 3.

“Our allowance is very small, and yet again it delays.” FGD 4.

### Relationship with undersigned private units

Some private units don’t turn in data to the HC. This means the population proportion that seeks health services from them is not represented in national datasets.

“There are many private HCs that don’t report.”KII6.

“Some health structures operate illegally so we can’t collect their data” KII4.

### Recruitment and training harmonization

The facility and respective implementing partners run parallel recruitment and training schedules. The health facility runs a recruitment and training policy under public health service. In the same regard, the implementing partners have internal human resource policies. Given the intersection of work and roles, discrepancies in renumeration across related job descriptions may kill the morale of underprivileged staff. “Implementing partners recruit data staff on short contract basis. So, the staff are not motivated after all the contracts are to expire soon.” KII5.

“We received training from Mildmay at Brovard Hotel, and from Rakai at the District.” KII2.

“Each time they bring a new trainer” KII6.

### Quality control and M&E

Quality control, and M&E protocols implemented by the facility are divergent from those implemented by partners. Similar data processes, managed by different service providers i.e., the HC and the implementation partners, may face different levels of scrutiny and support supervision, creating discrepancies and unconformities in would-be similar data “The implementing partner sets a target of like 10 positives per day.” KII 1.

“Funders demands(targets) give us so much pressure.” KII5.

“You may be pushed to force a positive to hit the targets” KII1.

### Procurement and supplies policies

Existing procurement and resource allocation policies don’t exploit health data thus hindering capacity building among staff. The National Medical Stores uses a push system for lower-level HCs. Therefore, procurement is not informed by the tediously collected data. The staff may also not feel responsible for supplies they didn’t order for leading to expiries.

“We make orders basing on the data we have but NMS (National Medical stores) and JMS (Joint Medical Stores) supply what they have.” KII 3.

“Allocation of resources is at a higher level. Therefore, knowing all this(having datasets) may not help the decisions that are made at a higher level.” KII 1.

### Data collection tools and related supplies

Shortages in tools and other supplies increase the data workload. Shortages in HIMS tools, meant improvision by the facility stuff, and double entry in the end thus making the whole process highly tedious.

“We occasionally run out of registers and we always improvise. When the registers come, those who can, transfer this information but this essentially doubles our workload.” (KII1).

“Sometimes they bring us the tools abruptly, they call us and then force us to work under extreme pressure.” FGD2.

### Interfacility collaboration

There is a window to share supplies between different HCs in the district. This could reduce the impact of delayed supplies from National Medical Stores(NMS).

“We borrow registers from other facilities and we refund them when our supplies arrive” KII5.

### Health data redundancy

There is a big time-lag between data collection and expected utilization due to facility insufficiencies in data analysis. The local relevance of the data is mostly lost to time. “There are quarterly assessments and comparisons.” KII6. “There’s no local analysis and therefore results are only relayed back after the district quarterly meetings.” KII1.

### Data dissemination funding

Community outreach programs that would aid dissemination to the community are underfunded.

”There is a plan to warn communities about seasonal diseases but it is not funded. The program is therefore implemented at just around 50%”. KII4.

### Community research feedback

VHTs, given their role as community health workers will most likely participate in many research projects in different roles. Failure to disseminate results or progress will leave VHTs exposed to the community wrath.

“Data is not given back to people by health organizations that collect it” FGD 1.

“We are not given feedback about the research projects we partake in.” FGD3.

“We are not given explanations for failed programs, so we also can’t explain to community members.” FGD3.

### Multidisciplinary community engagement

VHTs need support from healthcare workers during community engagement to diffuse the bias attached to VHTs.

“Relaying information to the public needs a new face, not only the usual VHTs” FGD3.

“People are so adopted to the VHTs that it is becoming hard to collect data” FGD3.

“We revisit the communities every after 3 months but people get tired of us” FGD2.

“Some people think we don’t have capacity to do our work” FGD2.

### Economic perceptions

Community members misconceive VHTs’ work as highly paying employment and they usually want a “share” of the earnings. So, community members are left with unmet economic expectations, which negatively affects the rapport built by the VHTs.

“Some community members think we are paid a lot of money and we don’t share with them.” FGD2.

“People think we are supposed to give them something each time we visit them, eventually they chase us since we don’t give anything.” FGD4.

### Political perceptions

Political affiliations influence community health data collection and dissemination. VHTs are resented upon political differences especially in political seasons.

“Some community members assume that we are campaigning for the government so they don’t receive us well” FGD1.

“Some people think we have political intentions so they don’t welcome us” FGD2.

#### Themes, codes and supporting verbatim Table 2

## Discussion

### Facility Staff and VHTs are positively receptive of an optimal health data management and utilization process and underscore the value therein

We discovered that, healthcare workers perceive an optimal data collection routine and process positively just as in studies carried out in Ethiopia, Tanzania and Kenya [22, 31, 40]. Their attitudes dwindle upon prevailing discrepancies associated with the public health service and current HMIS which they yearn for a solution. There is a possibility that the data collection outset in practice discourages determined healthcare workers.

This further asserts that that optimal HMIS/EMRS data management and utilization is mostly negated by systemic factors instead of personal factors [1, 3, 5, 23].

### The facility needs an enabling environment in terms of space, equipment, staffing and renumeration

The findings reaffirmed that most rural lower HCs in LICs like Mirambi HC III lack the basic structural and operational capacity, more so in the aspect of data and records management [36, 4]. Critical understaffing leaves healthcare staff overwhelmed. They therefore render less focus to authentic data collection and handling. Lack of Computerized data handling system has also predisposed the collected data to avoidable losses to rain, parasites, omission, and disintegration. The supporting VHTs are not renumerated at all and find themselves less motivated to collect community data [29]. Final datasets may have highly misleading data that may not facilitate reliable data-based decision making [10]. This also misrepresents the health picture of the community the facility serves. Introduction of a computerized system and recruitment of critical staff will improve efficiency, safety and quality of patient care [14, 33].

### There is a need to develop and implement a robust, elaborate and standard training module in the core aspects of data management (collection, handling and utilization) for the data focal personnel and other healthcare staff

Healthcare personnel and data staff explicitly agitated for routine, elaborate training in the data flow chain starting from data collection, but most importantly in analysis and utilization. Limited history of ICT training, and subsequent non-exposure to Information Technology (IT) tools has interfaced with rapidly evolving healthcare ICT trends [37]. Healthcare staff admitted that the training they received was insufficient just as in Malawi [3], but were more worrisome of the 2 weeks certification for previously naïve data handlers.

Health data becomes useless if key facility staff can’t translate it into functional units. Healthcare workers need capacity to ensure authenticity of data so they can plan according to the documented consumption and output of their respective departments [9, 20]. The health data focal personnel should have analytical skills to help deliver this key information Training health workers has improved data management endpoints in studies [30].

### Develop quality control, and M&E protocols but tailor them to the facility needs and output

We discovered a conspicuous omission of data quality control, and standardized, practical Monitoring and Evaluation (M&E) just as Asiimwe, Mboera and Mazengia [5, 22, 23] did. This is not only a standalone problem, but also a compounded effect from other challenges at the facility. The facility didn’t have a standing in-charge, and therefore lacked a supervisory figure.

There is a need to generate or adopt, execute and update standing health data management Standard Operating Procedures (SOPs) at facility level. These ought to be embedded into routine clinical service execution for one or more of the following reasons:

First, being a repetitive process, the data chain ought to have checks and balances at every critical stage to ensure uniformity.

Secondly, given the rapid evolution of technologies in healthcare [15], and presence of multiple support and implementing partners, there is an urgent need for uniform, clear data quality standards of optimally applicable value [45].

Thirdly, certain quantitative measurements could be misleading if not well elaborated. This involves most of the routine service provision targets like, positive Rapid Diagnostic Tests (RDTs) per day/week. Data can be fabricated to please funders or supervisors hence the need to review standing M&E protocols.

### The facility would benefit from procurement and supply policies that promote and utilize the aspects of optimal routine data management and utilization

We discovered that the facility feeds from the push supply system. However, this has left a string of bad luck. First, the healthcare workers are discouraged from using the data they collect, and so they lose the purpose of data collection. Secondly, this system doesn’t build capacity to translate data into workable plans. Thirdly, it has created shortages in the data chain its self, from registers to field gear used by VHTs, further impacting data management negatively. More severely, it has created shortage of critical medical supplies, undercutting healthcare efforts.

Healthcare delivery is evolving towards personalization. This caters for both the needs of individual community members, individual communities, and thus individual HCs. For this to be achieved, the individual, community and facility needs ought to be quantified from available data [35]. This means procurement should be based on the dynamic quantified needs of the facility rather that rationed proportions [39, 41]. Given the current delays in the data chain, supplies may serve lapsed demand [19].

Bottom-top approach to planning, backed by authentic, well managed data would not only optimize the demand–supply chain, but would also build capacity in facility staff, and create a sense of responsibility over supplies thus reducing shortages and wastage.

### There is a need for improved coordination between the facility and its implementation partners to create harmony in health data management

Our findings reveal a need for harmonized recruitment and training of staff, supervision, M&E and quality control in health data across the service provision spectrum. Different trainers from different implementing partners may only cause confusion and mayhem. It was discovered that different implementing partners operate different lines of care at different intensities respectively. However, there is a significant degree of overlap across the different clinical services. Multiple poorly coordinated subsystems may ambiguate data reporting demands, overwhelm and overburden facility staff, create omissions and duplicity and disconnect related facility departments [45, 18, 34]. Subsequently, this injures the quality, integrity and reliability of data.

The HC III also oversees the entire subcounty, and is mandated to aggregate data from the implementing partners, and private practitioners into the district datasets. If private facilities data is missed, then the healthcare picture of the community is incomplete [5].

### Reduction in information relay time, and improved community engagement will optimize the impact of health data utilization

We discovered that analysis is earmarked for the DHIS. But data analyzed locally would best suit facility needs. It also brings an advantage of easy, timely and convenient accessibility thus timely utilization. Timely availability of analyzed data would create a timely basis of action during routine clinical care and public health surveillance [14, 33, 45].

Coupled with enhanced community engagement, improved health information and services uptake would be realized hence an amplified data utilization impact. VHTs advanced the idea of involving facility staff in community engagement to improve the reception of health information in the community and countering political and economic biases associated with VHTs.

## Conclusions

Our findings reveal a very urgent need to build optimal routine health data management capacity and resilience in lower-level HCs in order to give a lifeline to clinical and public health services offered to the respective communities. We discovered that HCP’s enthusiasm in health data is dwarfed by a range of systemic challenges such as understaffing, poor skills, lack of tools and equipment, limited funding, lack of quality assurance, political differences, poor coordination and unfavorable policies. In the end, we have data of poor quality that is misrepresentative, non-utilizable and underutilized.

Building analysis capacity at facilities will allow prompt utilization of generated data thus smoothen flow of services. DHISII data is very big, less structured and heterogenous, hard to model for individual facility/community-based interventions, monitor their progress, outcome and impact. Each community has unique and diverse challenges that can only be solved through having a prompt outlook on the presentation of individual patient and public health records at the health facilities [7].

Installing IT capacity at the facilities will avail secure and easily retrievable storage means for individual patient records and foster personalized patient healthcare [7]. Healthcare workers will have to be equipment with the necessary IT knowledge so they could own the health data, ensure its accuracy and authenticity. This will also lay a strong foundation for future EMRS roll out.

Improving the working conditions of VHTs will ease their work, increase their enthusiasm and cause a positive impact on community data collection and information dissemination [29].

Improved coordination between facilities and implementing partners in the field of health data will create uniformity in the health data arena.

Lastly, adoption of the pull supply system at facilities will promote health data utilization in planning.

LICs are health resource limited settings. The best way to optimize the impact of limited resources is proper utilization of authentic data. This would help accurately determine problems and priority needs, pinpoint interventions, track their progress, outcome and impact.

## Data Availability

The datasets generated/analysed are not available publicly, but could be considerably shared upon request.

## Abbreviations

DHIS: District Health Information System
DHO: District Health Office
EDR: Electronic Data Record
EMRS: Electronic Medical Records System
FGD: Focus Group Discussion
HEPI: Health Professional Education and Training for Strengthening the Health System and Services in Uganda
HIV: Human Immunodeficiency Virus
HMIS: Health Management Information System
ICT: Information Computer Technology
IRB: Institutional Review Board
IT: Information technology
KII: Key informant Interviews
LICs: Low Income Countries
M&E: Monitoring and Evaluation
MoH: Ministry of Health
NMS: National Medical Stores
RDTs: Rapid Diagnostic Tests
TB: Tuberculosis
VHT: Village Health Team
